# Mucinous cystadenoma of the caecum: a rare cause of recurrent intussusception in a 3-year-old boy (case report)

**DOI:** 10.11604/pamj.2024.48.81.44175

**Published:** 2024-07-01

**Authors:** Jessica Dei-Asamoa, Abigail Anima Owusu, Samira Abdulai, Samuel Essoun, Antoinette Bediako-Bowan, Benjamin Akinkang, Solomon Edward Quayson, Victor Etwire, Hope Glover-Addy, Afua Adwo Jectey Hesse

**Affiliations:** 1 Department of Surgery, Korle-Bu Teaching Hospital, Accra, Ghana,; 2 Department of Pathology, Korle-Bu Teaching Hospital, Accra, Ghana,; 3 Department of Surgery, University of Ghana Medical School, Accra, Ghana,; 4 Department of Pathology, University of Ghana Medical School, Accra, Ghana,; 5 Accra College of Medicine, Accra, Ghana

**Keywords:** Mucinous cystadenoma, intussusception, children, caecum, case report

## Abstract

Mucinous cystadenoma of the caecum is an exceptionally rare occurrence, particularly in paediatric patients. They have been rarely reported in the appendix, ovary, pancreas, and liver. This is the first report of a mucinous cystadenoma of the caecum (to the best of the authors’ knowledge) in a child. A mucinous cystadenoma of the caecum can serve as a pathological lead point in intussusception. We report a case of a 3-year-old boy with a mucinous cystadenoma of the caecum causing intussusception. The intussusception recurred after an initial successful hydrostatic reduction. He had a laparotomy which revealed a caecal mass for which a limited right hemicolectomy was done. The histological diagnosis of the caecal mass was a mucinous cystadenoma. In intussusception caused by a lead point like a mucinous cystadenoma, an enema reduction may be successful but the intussusception may recur. Physical examination may reveal pathological lead points not detected on ultrasound scans. This case report contributes to the limited literature on mucinous cystadenomas of the caecum and calls for the need for further research to better understand their aetiology, clinical manifestation, histopathological diagnosis, and management strategies.

## Introduction

Intussusception is the most common cause of intestinal obstruction in children aged 3 months to 6 years [1]. The aetiology of paediatric intussusception in over 90% of cases is idiopathic [2]. However, in about 10% of cases, a precipitating lesion, called a pathological lead point may be the cause of the intussusception. Some of these lead points include Meckel´s diverticula, polyps, duplication cysts, lymphomas, lipomas, haematomas, lymphangiomas, and haemangiomas [2]. A mucinous cystadenoma is a benign tumour that is more commonly found in the ovary, pancreas, or appendix and rarely in the lungs [3]. A mucinous cystadenoma of the ascending colon presenting as intussusception was reported as a novel presentation by Shaco-Levy *et al*. in 2003 in a 32-year-old woman [4]. There has been no report to the best of the authors´ knowledge of a mucinous cystadenoma of the caecum with or without an intussusception in a child. We report a rare finding of a mucinous cystadenoma of the caecum as a pathological lead point causing intussusception in a 3-year-old boy.

## Patient and observation

**Patient information:** a 3-year-old male presented with worsening intermittent abdominal pain and non-bilious vomiting of a week´s duration. He had no abdominal distension and no fever. He had no chronic illnesses and no past surgical interventions.

**Clinical findings:** on physical examination, he looked acutely ill, febrile (T-38.0°C), anicteric, not pale, and with dry oral mucosa. His abdomen was full, soft, and non-tender. There was no organomegaly, and no masses were palpated. His digital rectal examination was normal. His heart rate was 152 beats per minute (bpm). Other cardiovascular and respiratory system examinations were normal.

**Figure 1:**
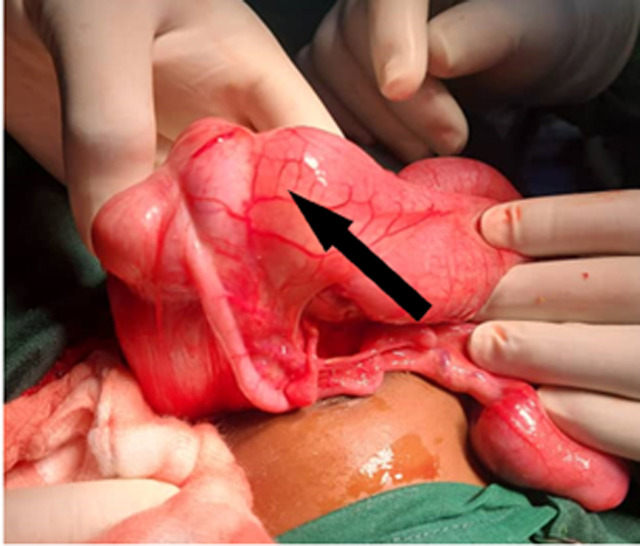
the caecum, appendix and ileocaecal junction of the child showing a tumour in the caecum jutting out into the ileum (arrow)

**Diagnostic assessment and therapeutic intervention:** an abdominal ultrasound scan was done which revealed a right upper quadrant mass with the target sign which was reported as intussusception. His white blood cell count (WBC) was 5.56x10^9^/L, hemoglobin (Hb) level was 10.5g/dl, and platelet count was 558 x 10^9^. His serum urea, electrolytes, and creatinine were normal. After a diagnosis of intussusception was made, he was given intravenous (IV) fluids, IV antibiotics, and paracetamol. He had a successful ultrasound-guided hydrostatic reduction (USGHR) of the intussusception which was evidenced by the flow of saline back into the small bowel. Two days after the USGHR of the intussusception, his symptoms recurred and a repeat ultrasound scan showed the target sign and pseudo kidney sign again, present in the mid-abdomen which was suggestive of a recurrent intussusception. He had a successful repeat of the USGHR. However, a mass was palpated in his right iliac fossa after the procedure. Based on this, a laparotomy was performed, and the findings were “a tumor in the caecum completely occluding the lumen and multiple enlarged mesenteric lymph nodes” (Figure 1). The patient had a limited right hemicolectomy and the specimen was sent for histopathology.

**Figure 2:**
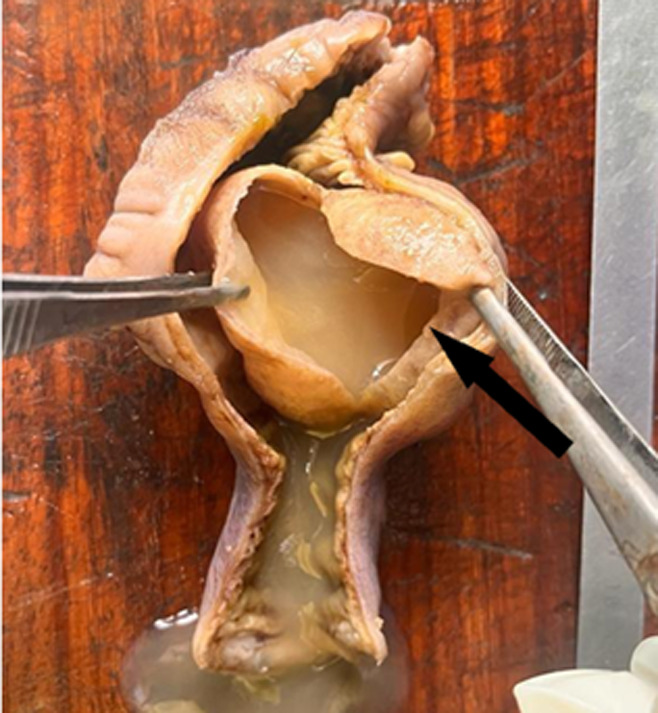
the open caecum showing an uninoculated cyst containing cream mucoid fluid

**Figure 3:**
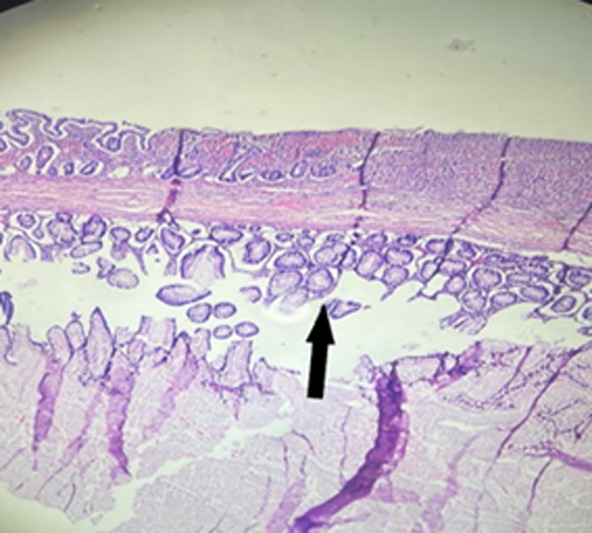
photomicrograph of a section of the caecum showing a cyst lined by mucin-producing columnar epithelial cells (arrow)

The gross pathological examination of the mass revealed a well-circumscribed tan, soft, cystic lesion measuring 70x50x40mm completely occluding the large bowel at the site of distension. The lesion was adherent to parts of the mucosa of the caecum but was detached in some areas. The cut surface of the lesion showed an uniloculated cyst containing off-white mucoid fluid. The inner lining of the cyst was smooth and shiny. The cyst wall was 2mm thick. The rest of the resected small and large bowel was grossly unremarkable (Figure 2). Microscopic evaluation showed “the cyst lined by mucin-producing columnar epithelial cells with basally located nuclei without atypia with lamina propria containing few glands and moderate mixed inflammatory cells. The external surface of the cyst was covered by colonic mucosa with heavy mixed inflammatory infiltrates and foci of ulceration. The remaining adjacent bowel wall and appendix were histologically normal. No features suggesting malignancy were seen” (Figure 3). A diagnosis of mucinous cystadenoma was therefore made.

**Follow-up outcomes:** the postoperative period was uneventful. He was discharged home on postoperative day 4 and reviewed at the clinic on postoperative days 13 and 55. He is doing well. He will be followed up with a colonoscopy at 6 months post-surgery, then 3 yearly for two to three times. If these are normal, he will have colonoscopies every 5 to 10 years.

**Informed consent:** written consent was given for this publication by the patient´s guardian.

**Patient´s perspective:** the patient´s guardian thinks they received adequate treatment and they were happy with his recovery.

## Discussion

Mucinous cystadenoma is a benign tumour found in the ovaries of women of childbearing age but may also be found in other organs such as the pancreas, appendix, liver, lungs, and ascending colon [3-5]. Its presence in the caecum acted as a pathological lead point and the cause of the recurrence of the intussusception 2 days after a successful reduction. A close differential for mucinous cystadenoma of the colon is colitis cystica profunda which is a rare benign lesion of uncertain aetiology composed of mucous-filled cysts in the wall of the colon or rectum. Histologically the submucosa is widened due to the presence of mucous-containing cysts which may involve the muscularis propria and serosa [6]. Mucinous cystadenoma of the bowel should be distinguished from mucinous cystadenocarcinoma which is a malignant neoplasm. Imaging modalities such as the computerized tomographic (CT) scan, may show the presence of septations, locations, and solid areas within the cyst and calcifications in the wall suggesting malignancy [5]. Regardless of the advances in imaging technology, to make a definitive (gold standard) diagnosis of mucinous cystadenomas, a histopathological examination of the surgical specimen must be done [6].

Despite delayed presentation, there is a 75% success rate of USGHR for intussusception at the Korle-Bu Teaching Hospital, a tertiary facility in Ghana [7]. Most attempts at hydrostatic reduction are successful as was seen in this case. The recurrence rate of intussusception is up to 20% and the presence of a pathological lead point significantly increases the risk [8]. The lead points commonly encountered are Meckel´s diverticula, intestinal duplications, polyps, and lymphoma [2]. Pathological lead points have been reported to be more common in adults and older children and less common (10%) in the age category this child fell into [2].

Despite the high sensitivity and specificity of ultrasound scans for the diagnosis of intussusception, they rarely detect pathological lead points [9]. It is therefore not surprising that the mass was missed on both ultrasound scans as a pathological lead point and described as only an intussusception. Abdominal examination during hydrostatic reduction, while the patient is sedated and not resisting examination, is a useful adjunct in the detection of pathological lead points in intussusception. There are currently no guidelines on the follow-up of children with mucinous cystadenoma of the bowel, which could be due to the rarity of the disease. The researchers however recommend a colonoscopy at 6 months after surgery to rule out synchronous lesions and then 3 yearly for two to three times. If normal, a colonoscopy every 5 to 10 years is recommended. This is based on guidelines on the follow-up of children with a high risk of developing colonic tumours [10].

## Conclusion

Mucinous cystadenomas can occur in the caecum and they can be a rare cause of recurrent intussusception. In this case, the diagnosis is unusual not only regarding the location but also because of the patient's age (3 years). Ultrasound scans are highly sensitive and specific in the diagnosis of intussusception but may not detect pathological lead points. Physical examination of the child, while sedated, serves as a useful adjunct in the diagnosis of pathological lead points and is highly recommended. This case report contributes to the limited literature on mucinous cystadenomas of the caecum, emphasizing the importance of further research to better understand their aetiology, clinical manifestation, histopathological diagnosis, and management strategies.

## References

[1] Waseem M, Rosenberg HK (2008). Intussusception. Pediatr Emerg Care.

[2] Cera SM (2008). Intestinal intussusception. Clin Colon Rectal Surg.

[3] Luo ZY, Shen XZ, Liu F, Lin C (2021). Pulmonary mucinous cystadenoma complicated with infection: A rare case report. Medicine.

[4] Shaco-Levy R, Tsodikov V, Levy I (2003). Mucinous cystadenoma of the ascending colon: a novel presentation. Scand J Gastroenterol.

[5] Liang GB, Lu YP, Huang XK, Shi M (2009). Images for diagnosis: Primary mucinous cystadenoma of the ileum. Chin Med J (Engl).

[6] Krummel TM, Bell S, Kodroff MB, Berman WF, Salzberg AM (1983). Colitis cystica profunda: A pediatric case report. J Pediatr Surg.

[7] Mensah Y, Glover-Addy H, Etwire V, Appeadu-Mensah W, Twum M (2011). Ultrasound guided hydrostatic reduction of intussusception in children at Korle Bu Teaching Hospital: an initial experience. Ghana Med J.

[8] Ye X, Tang R, Chen S, Lin Z, Zhu J (2019). Risk factors for recurrent intussusception in children: A systematic review and meta-analysis. Front Pediatr.

[9] Munden MM, Bruzzi JF, Coley BD, Munden RF (2007). Sonography of pediatric small-bowel intussusception: Differentiating surgical from nonsurgical cases. American Journal of Roentgenology.

[10] Barnard J (2009). Screening and surveillance recommendations for pediatric gastrointestinal polyposis syndromes. J Pediatr Gastroenterol Nutr.

